# Placentas From COVID-19-Positive Mothers: A Morphological Study of 20 Cases in Marrakesh, Morocco

**DOI:** 10.7759/cureus.107176

**Published:** 2026-04-16

**Authors:** Hind Rachidi, Hanane Rais, Bouchra Fakhir, Nabila Soraa, Fatima-Ezzahra Hazmiri

**Affiliations:** 1 Laboratory of Morphoscience Research, Department of Pathology, Faculty of Medicine and Pharmacy, Cadi Ayyad University, Mohammed VI University Hospital, Marrakesh, MAR; 2 Laboratory of Infectious Disease Research, Department of Microbiology, Faculty of Medicine and Pharmacy, Cadi Ayyad University, Mohammed VI University Hospital, Marrakesh, MAR

**Keywords:** fetal origin, hypoxia-ischemia, inflammatory pathology, maternal origin, placental vascular malperfusion, sars-cov-2

## Abstract

Background

Due to COVID-19, hospitals have seen a large influx of COVID-19-positive pregnant women. In order to study the impact of this virus on the infant and the possibility of its vertical transmission, we led a morphological study of 20 placentas from women infected or with a history of SARS-CoV-2 infection during pregnancy.

Objective

The aim of our study was to present the morphological characteristics of the placentas from these women and to correlate them with the clinical and biological findings, highlighting their relevance to pregnancy outcome and vertical transmission.

Study design

This is a retrospective, observational study, carried out at the pathology department of Mohammed VI University Hospital in Marrakesh over a one-year period from June 2020 to June 2021. The detection of SARS-CoV-2 by reverse transcription (RT)-PCR was carried out on the placental tissue and the newborns' urine and stools. Clinical information was taken from the medical records of patients in the gynecology and obstetrics department.

The placentas received in our department were described and sampled according to the Amsterdam consensus.

Results

The mean age of the mothers was 29.85 years. Only one of them (1/20) had a history of cesarean section for severe preeclampsia. Thirteen (13/20) women had a positive nasopharyngeal PCR and were symptomatic at admission. Two critical states were noted: one case of severe preeclampsia complicated by hemolysis, elevated liver enzymes, and low platelet count (HELLP) syndrome and another case of acute respiratory distress. Eight (8/20) deliveries were vaginally made and 12 (12/20) by cesarean section. The middle term of pregnancy was 36.7 weeks. Three cases of fetal death in utero (FDIU) were noted. There were no maternal deaths. The gross examination of our placentas revealed lesions in 18/20 cases. Placental vascular malperfusion lesions of maternal origin were predominant (16/20). Among these lesions, accelerated villous maturation (AVM) was found in 12/20 cases, central intervillous thrombosis (IVT) in 9/20 cases, and subchorionic intervillous thrombosis in 8/20 cases. Villous infarct lesions were found in 7/20 cases and were associated with a decidual arteriopathy in two cases. Placental vascular malperfusion lesions of fetal origin were found in 6/20 cases, with association to lesions of maternal origin in two cases. They all showed fetal vascular network lesions. The Inflammatory placental pathology was observed in only 4/20 cases. It was about one case of subchorionitis and three cases of acute chorioamnionitis associated with acute funiculitis. Massive perivillous fibrinoid deposition was found in 1/20 cases. The three fetal death in utero placentas were characterized by lesion multiplicity. Hypoxia-ischemia villous lesions were found in all of our placentas. Three newborns of positive mothers tested positive for SARS-CoV-2 RT-PCRs in their urine and stools. The corresponding placentas were also affected, including the one from the mother with HELLP syndrome.

Conclusion

SARS-CoV-2 Infection has no pathognomonic placental histopathological lesions. High rates of maternal origin lesions are reported in our study, suggesting abnormal maternal circulation. These results are generally concordant with those of the literature and justify close pregnancy monitoring.

## Introduction

The coronavirus (SARS-CoV-2) is an enveloped RNA virus belonging to the Coronaviridae family. It is responsible for the COVID-19 pandemic, initially identified in Wuhan, China [1], and characterized by high transmissibility [2]. As a result, hospitals have seen a large influx of COVID-19-positive pregnant women. The impact of this virus on pregnant women and infants is currently a key area of interest [3].

The possibility of the vertical transmission of the coronavirus and pregnancy complications is now serious concerns for these infected pregnant women [4].

The currently published studies have mainly focused on clinical and biological data in these women, who do not appear to be at a higher risk of severe disease compared to SARS-CoV or MERS-CoV infections. However, unfavorable perinatal outcomes have been reported, including an increased risk of miscarriage, preeclampsia, preterm delivery, and intrauterine fetal death (IUFD) [2].

Only three cases were reported in the first study at the beginning of the pandemic. The reported findings were not specific, including varying degrees of increase in perivillous fibrin and focal increase in syncytial nodes. One placenta showed a massive infarct, and a chorangioma was present in another one [5,6].

An English study series of 16 cases of placental pathology linked to COVID-19 did not report any pathognomonic morphological features. However, it demonstrated a high rate of maternal vascular malperfusion (MVM) injury and intervillous thrombosis (IVT), suggesting an abnormal maternal circulation, as well as an increased incidence of chorangiosis [2].

This study aimed to analyze the morphological features of 20 placentas from women with current or past SARS-CoV-2 infection during pregnancy; to correlate these findings with clinical and biological data from affected mothers and their newborns, with particular emphasis on pregnancy outcome and the risk of vertical transmission; and to compare the results with those reported in the literature.

## Materials and methods

Study design and setting

This was a retrospective, descriptive study conducted on a series of 20 placentas. The study was carried out in the pathology department, in collaboration with the gynecology and obstetrics department at the Mother and Child Hospital of Mohammed VI University Hospital in Marrakech, over a one-year period from June 2020 to June 2021.

Inclusion criteria

The study included the following: placentas collected from pregnant women admitted to the gynecology and obstetrics department at Mohammed VI University Hospital, as well as from private healthcare facilities, between 2020 and 2021; placentas from pregnant women who were infected with SARS-CoV-2 or who had a documented history of SARS-CoV-2 infection during pregnancy; and all placentas collected after obtaining verbal informed consent from the patients.

Exclusion criteria

The following were excluded from the study: placentas from patients who had been infected with SARS-CoV-2 prior to conception and placentas from patients who developed SARS-CoV-2 infection exclusively during the immediate postpartum period.

Data sources

Clinical data were retrospectively collected from patients' medical records in the gynecology and obstetrics department. Maternal data included age, obstetric history, comorbidities, COVID-19-related symptoms, and pregnancy complications. Neonatal data included gestational age at delivery, the mode of delivery, birth weight, the Apgar score, and neonatal outcomes.

Macroscopic and microscopic placental findings were retrieved from pathology reports and recorded using a standardized data collection form based on the Amsterdam Consensus [5] and practical fetal and placental pathology [7].

Laboratory and pathological methods

Maternal SARS-CoV-2 infection was confirmed by reverse transcription (RT)-PCR on nasopharyngeal swabs, and viral detection was also performed on placental tissue, as well as neonatal urine and stool samples collected within the first days of life under sterile conditions. Placentas were fixed in 10% buffered formalin for 24-48 hours and examined macroscopically, including the assessment of weight, dimensions, cord insertion, membranes, and any gross lesions. Representative samples were taken from maternal and fetal surfaces, including central and peripheral regions.

Tissue samples were processed using a Leica ASP300 S (Nussloch, Germany) vacuum infiltration tissue processor, embedded in paraffin, sectioned into 3 µm-thick slices using a semiautomatic Leica RM2245 microtome, and routinely stained with hematoxylin and eosin (H&E). The final number of slides obtained per placenta ranged from 10 to 20. Placental lesions were classified into four main groups: maternal vascular malperfusion (MVM), fetal vascular malperfusion (FVM), inflammatory lesions, and other lesions, according to the Amsterdam Placental Workshop Group Consensus [5].

## Results

Twenty placentas from mothers infected with SARS-CoV-2 or with a SARS-CoV-2 infection history during pregnancy were examined. The mean age of these mothers was 29.85 years, with extremes ranging from 18 years to 43 years. Only one patient had a history of cesarean section for severe preeclampsia; the others (95%) had no medical history, especially no chronic hypertension, no diabetes, and no heart disease. All infected women (65%) were admitted with flu-like illness consisting of a cold, headache, cough, sometimes a fever, and dyspnea. Two critical states were noted: The first one was a case of severe preeclampsia complicated by hemolysis, elevated liver enzymes, and low platelet count (HELLP) syndrome, and the second one was about an acute respiratory distress case that required intensive medical care. Nasopharyngeal PCR in these symptomatic patients was positive.

Forty percent of deliveries were vaginally made. Cesarean section was performed in 60% of cases, respectively, for acute fetal distress (AFD) (76%), preeclampsia (8%), threatened preterm labor (TPL) (8%), and transverse presentation (8%).

The mean gestational age of pregnancy was 36.7 weeks of amenorrhea (24-41); the majority of cases (80%) presented at a 37-41-week interval. Three cases of fetal death in utero (FDIU) (15%) were noted: one from a preeclampsia case at 24 weeks and two cases at 37 weeks with no associated clinical signs. There were no maternal deaths. The middle interval between SARS-CoV-2 infection diagnosis and delivery was 33 days (Table 1).

**Table 1:** Clinical and PCR findings AFD, acute fetal distress; DFIU, fetal death in utero; HELLP, hemolysis, elevated liver enzymes, and low platelet count; w, week; d, day

Case	Maternal age (year)	Gestational age (week)	Maternal medical history	Delivery	Infection-delivery interval (days)	Maternal nasopharyngeal PCR	Placental tissue PCR	Newborns urine and stool PCR
1	19	28 w + 3 d	-	Cesarean delivery (AFD)	6	(+)	(-)	(-)
2	18	32 w + 2 d	-	Cesarean delivery (HELLP syndrome + severe preeclampsia)	7	(+)	(+)	(+)
3	28	39 w	-	Vaginal delivery	65	(-)	(-)	(-)
4	34	39 w	-	Cesarean delivery (AFD)	4	(+)	(-)	(-)
5	26	36 w + 3 d	-	Cesarean delivery (AFD)	6	(+)	(-)	(-)
6	25	40 w	-	Cesarean delivery (AFD) (transverse presentation)	27	(-)	(-)	(-)
7	29	40 w + 1 d	-	Vaginal delivery	180	(-)	(-)	(-)
8	35	40 w + 1 d	-	Vaginal delivery	4	(+)	(-)	(-)
9	30	37 w	-	Vaginal delivery (FDIU)	12	(+)	(-)	(-)
10	28	41 w	-	Cesarean delivery (AFD)	7	(+)	(+)	(+)
11	37	39 w + 4 d	-	Cesarean delivery (AFD)	33	(-)	(-)	(-)
12	35	40 w	-	Cesarean delivery (AFD)	9	(+)	(-)	(-)
13	28	39 w	-	Vaginal delivery	2	(+)	(+)	(+)
14	23	40 w	-	Vaginal delivery	40	(-)	(-)	(-)
15	26	37 w + 4 d	-	Cesarean delivery (AFD)	3	(+)	(-)	(-)
16	33	39 w + 4 d	-	Cesarean delivery (AFD)	11	(+)	(-)	(-)
17	27	37 w + 3 d	-	Vaginal delivery (FDIU)	7	(+)	(-)	(-)
18	39	39 w	-	Cesarean delivery (AFD)	150	(-)	(-)	(-)
19	34	32 w	-	Vaginal delivery	60	(-)	(-)	(-)
20	43	24 w	Cesarean section for severe preeclampsia	Cesarean delivery (AFD) (preeclampsia) (FDIU)	21	-	-	-

**Figure 1:**
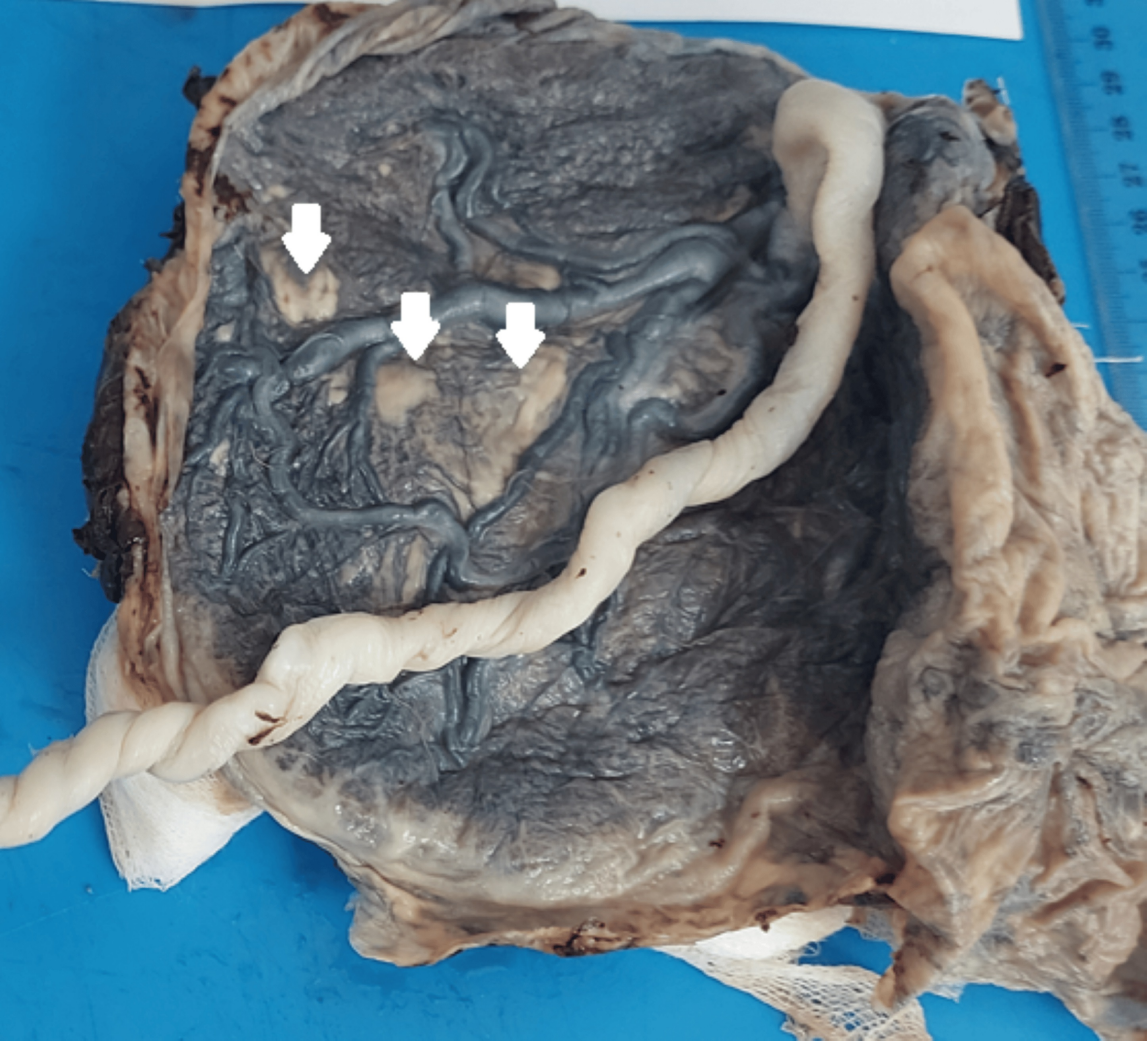
Microthrombosed placenta from an intrauterine fetal death (IUFD) showing three lesions of the chorionic plate (white arrows) measuring 1-2.3 cm (case number 9)

The gross examination of our placentas revealed lesions in 90% of cases. Forty-eight percent of these lesions were observed at the section slice, 32% in the chorionic plate, and 20% in the basal plate. The size of these lesions varies between 0.5 cm and 3.2 cm, with an average of 1.4 cm. An appearance of congestion and microthrombosed chorionic vessels was reported in one case (Figure 1).

According to the histological examination, the placental injuries were classified into maternal and fetal vascular malperfusion, inflammatory lesions, and others.

**Figure 2:**
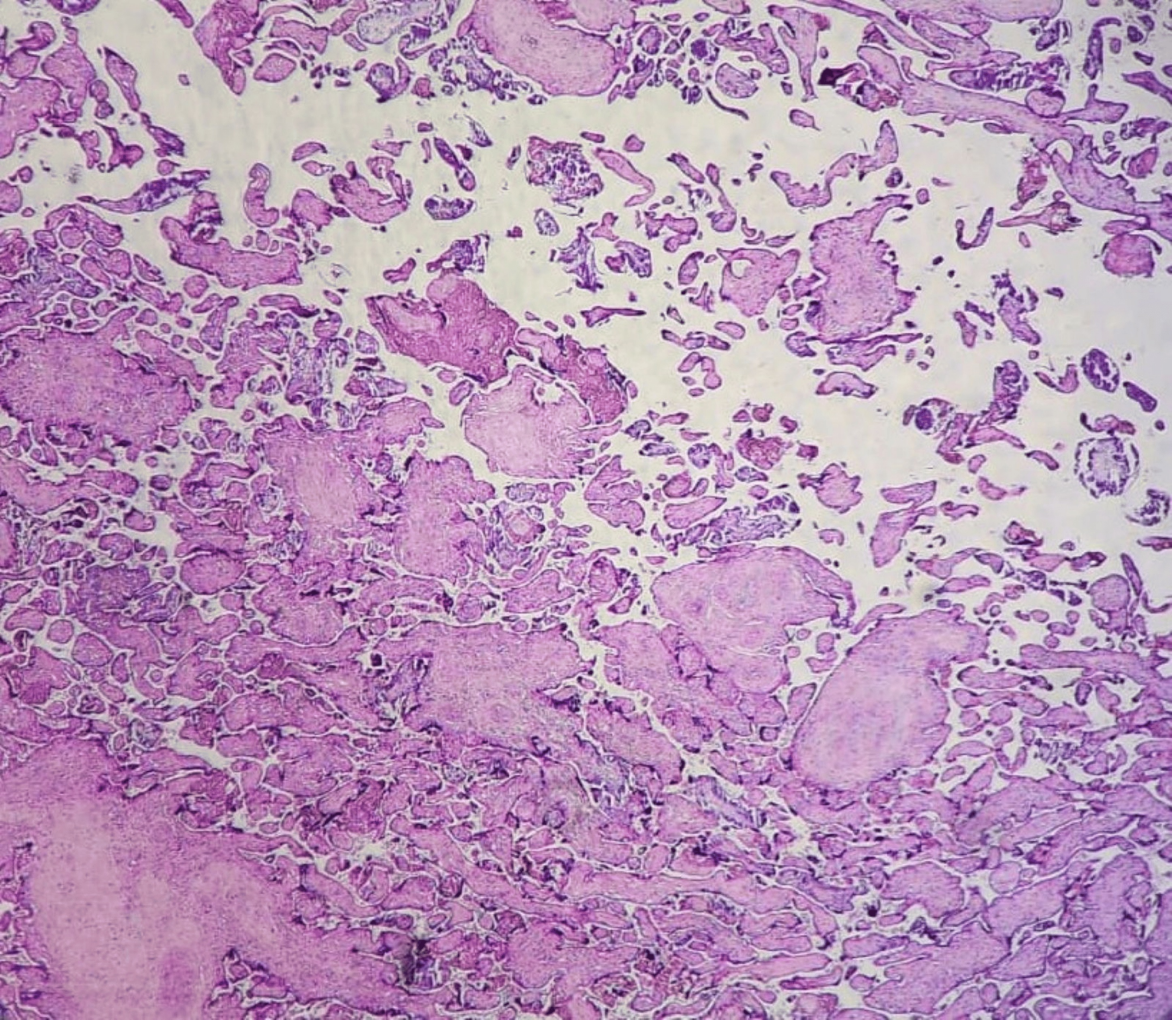
Accelerated villous maturation lesions in the placenta of a 28-year-old mother with no medical history (H&E staining, magnification ×4) (case number 10) H&E: hematoxylin and eosin

**Figure 3:**
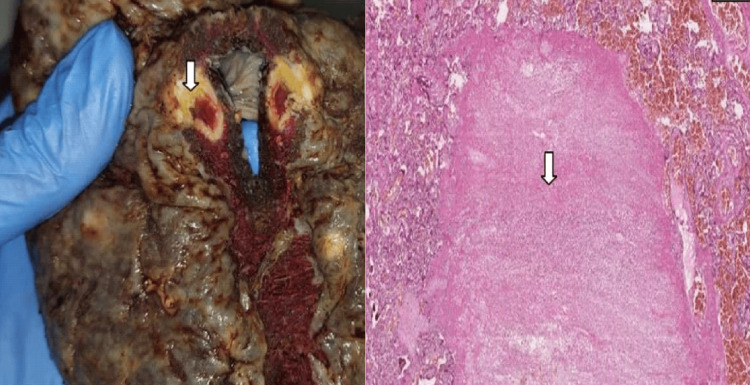
Heterogeneous lesion (white arrow) on the placenta section slice of an 18-year-old mother with HELLP syndrome, infected by the SARS-CoV-2 virus (left image) This lesion was histologically corresponding to a central intervillous thrombosis (right image) (H&E staining, magnification ×20) (case number 2) HELLP, hemolysis, elevated liver enzymes, and low platelet count; H&E, hematoxylin and eosin

**Figure 4:**
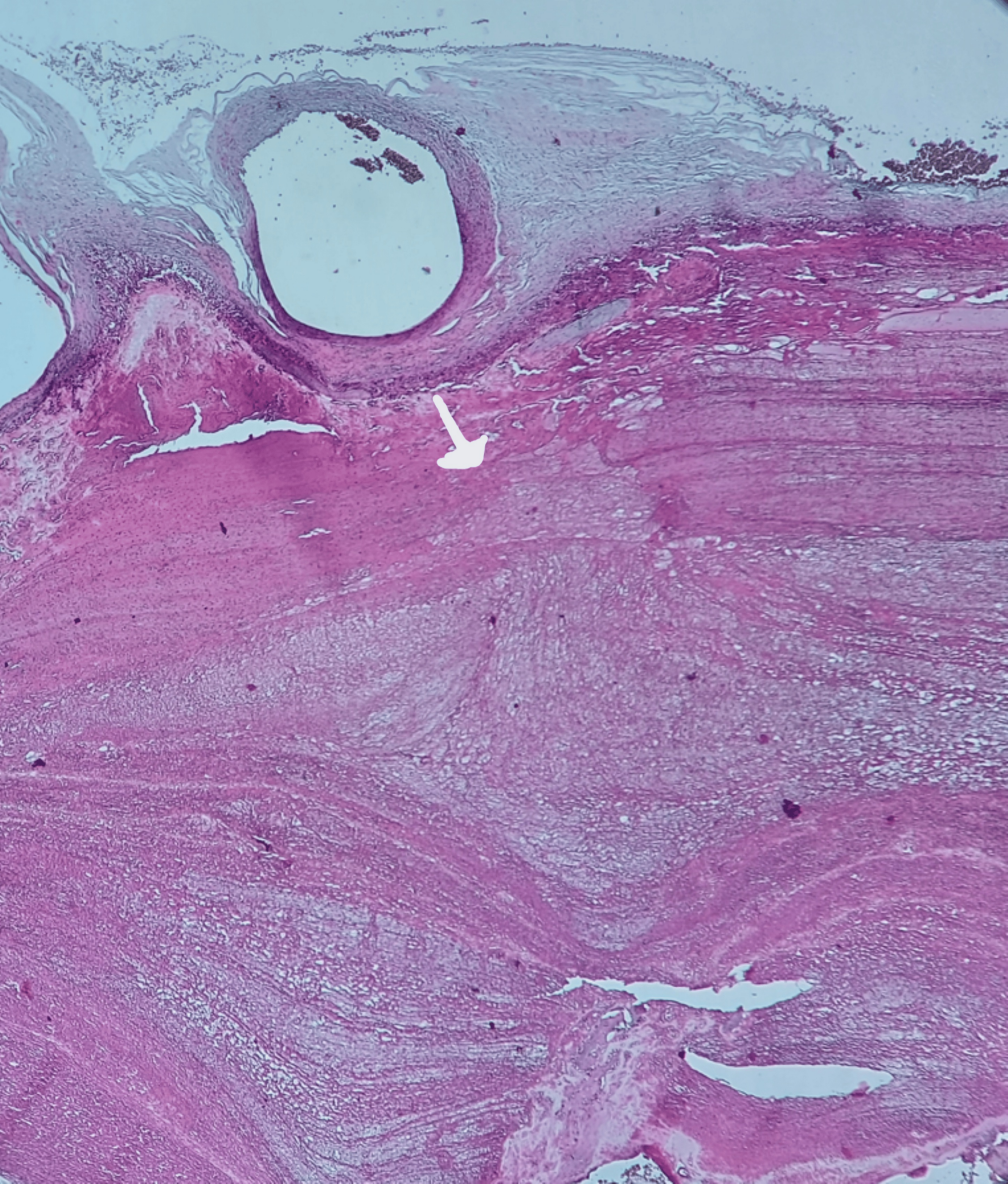
Subchorionic intervillous thrombosis (white arrow), pushing back the adjacent trophoblastic villi and containing parallel stratifications (Zahn lines) (H&E staining, magnification ×20) (case number 9) H&E: hematoxylin and eosin

**Figure 5:**
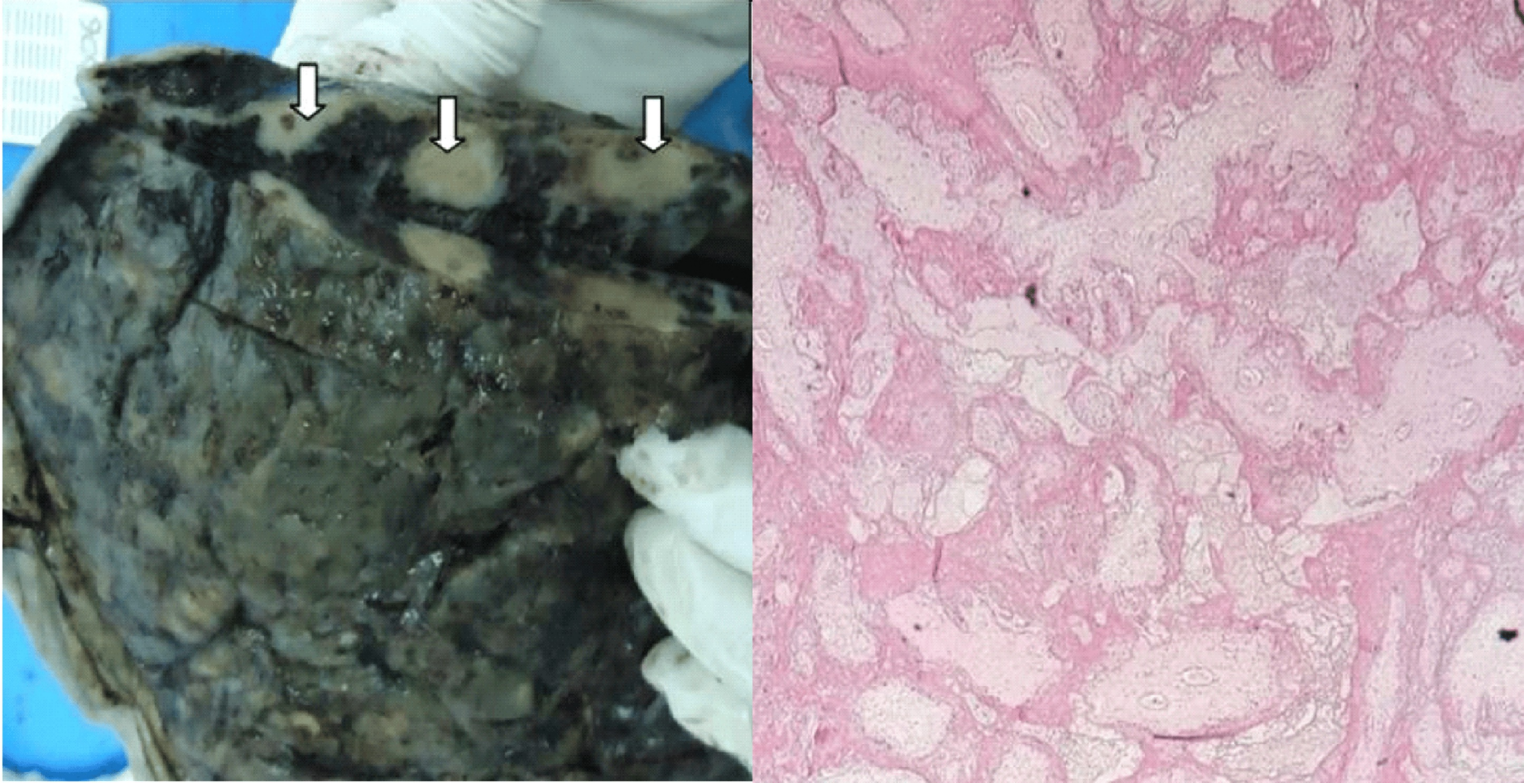
Three whitish lesions (white arrows) measuring 0.5-2 cm on a placenta section slice of the mother with HELLP syndrome (left image) These lesions correspond histologically to a central intervillous thrombosis (right image) (H&E staining, magnification ×20) (case number 2) HELLP, hemolysis, elevated liver enzymes, and low platelet count; H&E, hematoxylin and eosin

**Figure 6:**
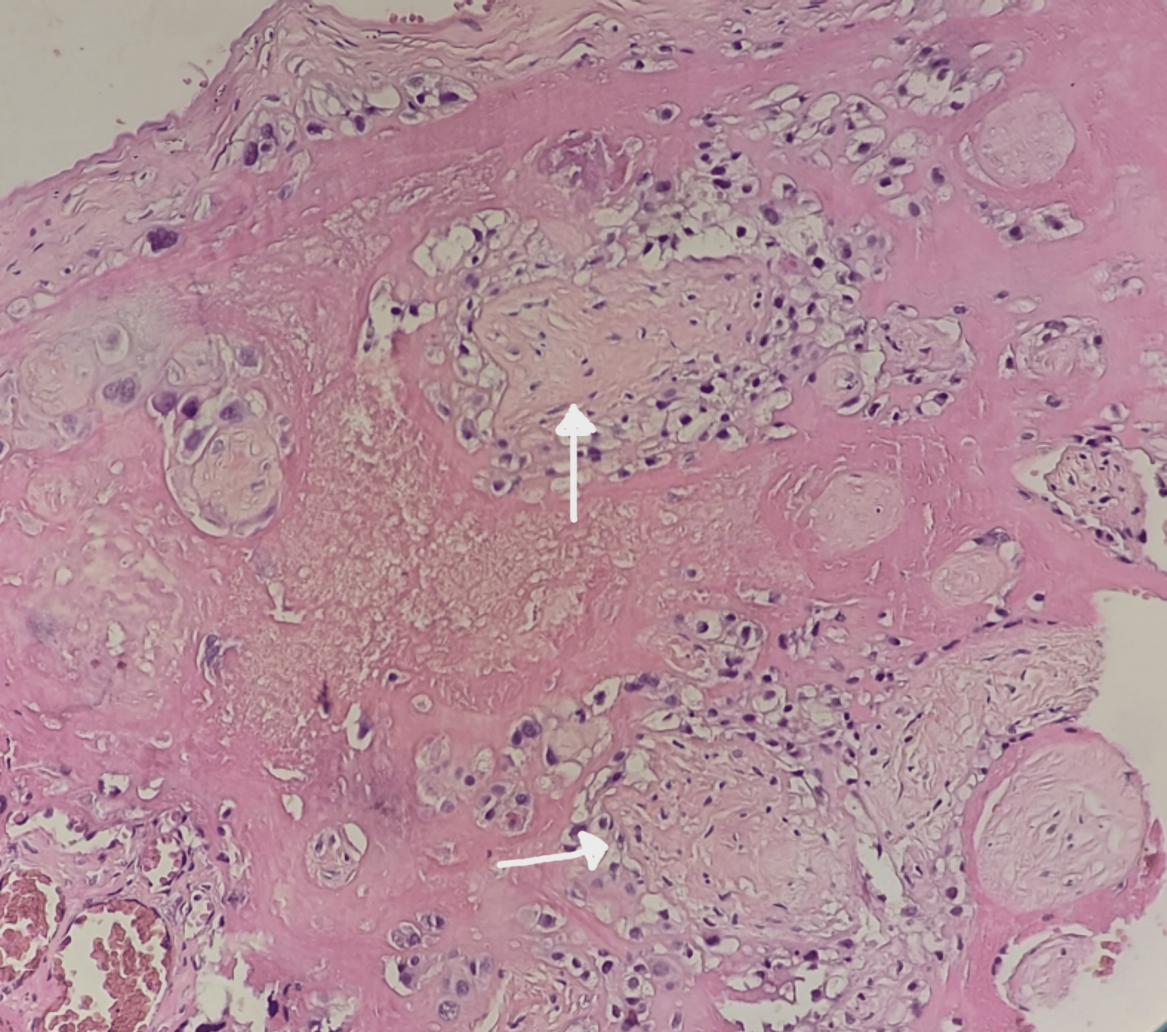
Decidual arteriopathy lesion (white arrows) in the placenta of the mother with HELLP syndrome (H&E staining, magnification ×40) (case number 2) HELLP, hemolysis, elevated liver enzymes, and low platelet count; H&E, hematoxylin and eosin

**Figure 7:**
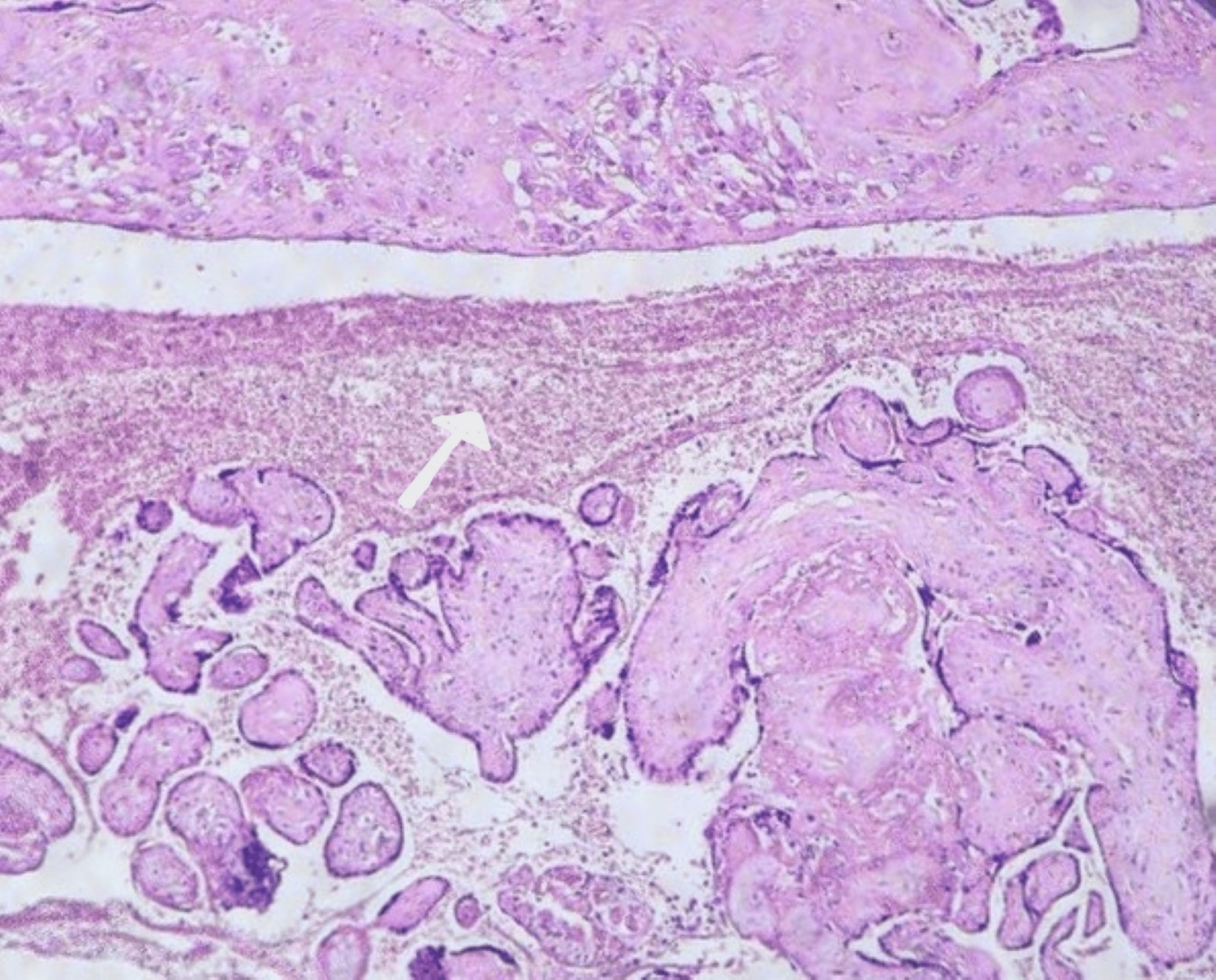
Basal decidual hematoma (white arrow) in the placenta of a mother with FDIU No maternal medical history was noted (H&E staining, magnification ×4) (case number 20) FDIU, fetal death in utero; H&E, hematoxylin and eosin

Placental vascular malperfusion lesions of maternal origin (MVM) were predominant (80%). Among these lesions, accelerated villous maturation (AVM) was found in 12 cases (60%) (Figure 2), central intervillous thrombosis in nine cases (45%) (Figure 3), and subchorionic intervillous thrombosis in eight cases (40%) (Figure 4). Villous infarct lesions were found in seven cases (35%). The lesion was central and isolated in three cases, marginal and isolated in two cases, and both and multiple in two cases (Figure 5). These last two cases (10%) had decidual arteriopathy lesions (Figure 6), associated with a basal decidual hematoma (retroplacental hematoma) in one of them (5%) (Figure 7).

**Figure 8:**
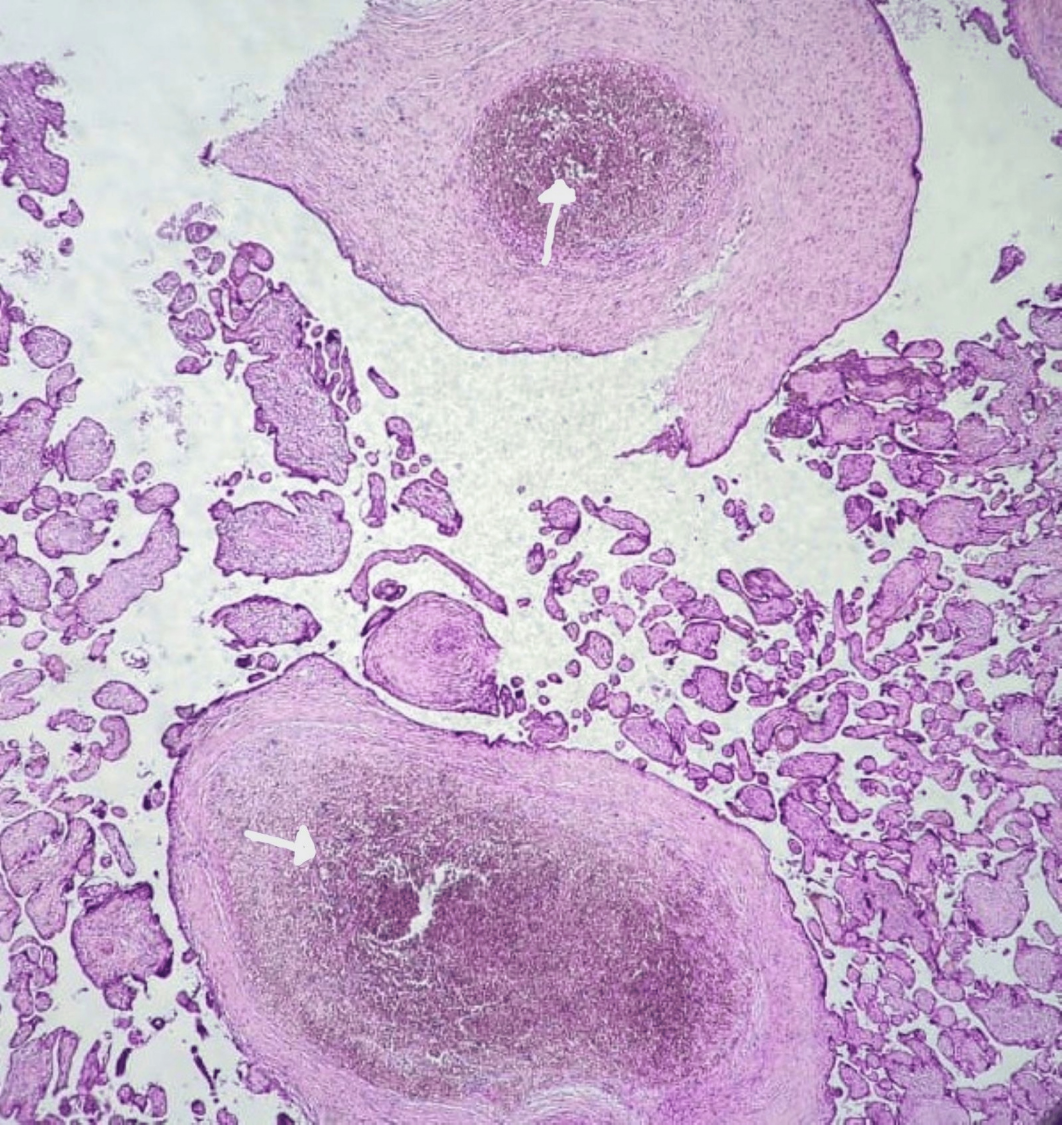
Chorioallantoic and stem vessel dilation (white arrows) associated with chorangiosis in a placenta with an FDIU (H&E staining, magnification ×4) (case number 9) FDIU, fetal death in utero; H&E, hematoxylin & eosin

Placental vascular malperfusion lesions of fetal origin (FVM) were found in 30% of cases (10% associated with MVM lesions). They all showed fetal vascular network lesions (Figure 8).

**Figure 9:**
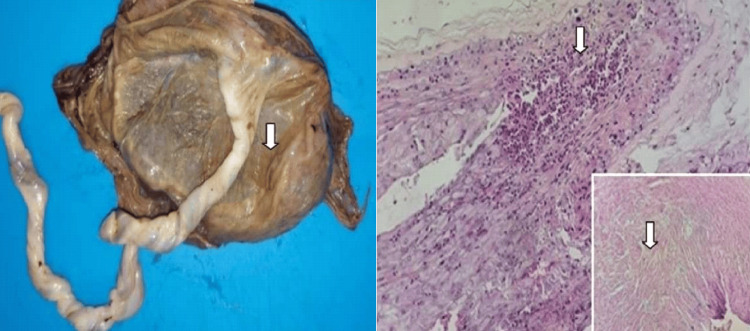
Macroscopic image (left image, white arrows) showing thickened, greenish free placental membranes in a placenta with stage 2 acute chorioamnionitis/funisitis and the corresponding histological examination (right image, white arrow) (H&E staining, magnification ×4) (case number 10) H&E: hematoxylin and eosin

The inflammatory placental pathology was observed in only four cases (20%), in association with MVM and FVM lesions. It was about a case of subchorionitis (5%) and three cases (15%) of acute chorioamnionitis associated with acute funiculitis (Figure 9).

**Figure 10:**
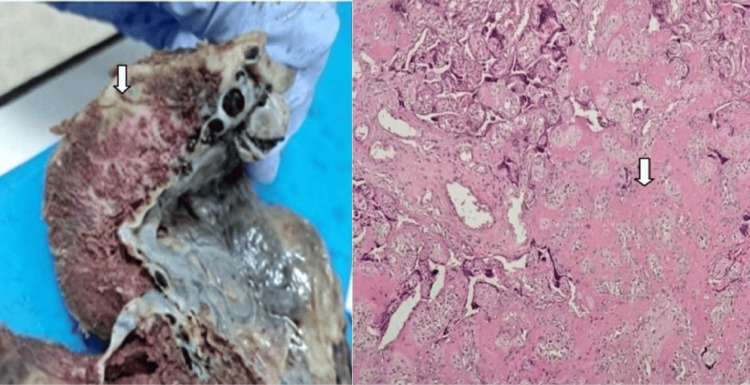
Macroscopic image (left image) showing a whitish, heterogeneous deposit in a placenta section slice, corresponding histologically (right image) to massive perivillous fibrinoid deposition (white arrow) (H&E staining, magnification ×4) (case number 10) H&E: hematoxylin and eosin

Massive perivillous fibrinoid depositions were found in one case (5%), in association with MVM lesions (Figure 10).

The three FDIU placentas were characterized by lesion multiplicity. The placenta from the woman with preeclampsia showed six infarcts (two central and four paracentral) measuring from 0.5 cm to 2.5 cm. These infarcts were associated with a basal decidual hematoma and decidual arteriopathy lesions in one case. The two other ones showed multiple subchorionic intervillous thrombosis associated with chorioallantoic vessels dilation (Table 2).

**Table 2:** Histopathological lesions of placentas

Lesions	1	2	3	4	5	6	7	8	9	10	11	12	13	14	15	16	17	18	19	20
Maternal vascular malperfusion																				
Decidual arteriopathy		X																		X
Accelerated villous maturation (AVM)	X					X	X	X		X		X	X	X	X	X		X	X	
Central villous infarct	X	X										X				X				X
Marginal villous infarct		X	X					X												X
Central intervillous thrombosis (IVT)	X	X									X		X		X	X		X	X	X
Subchorionic intervillous thrombosis (IVT)			X		X	X		X	X			X		X			X			
Basal decidual hematoma																				X
Fetal vascular malperfusion																				
Lesions of the fetal vascular network					X	X	X	X	X				X							
Inflammatory pathology																				
Maternal: chorioamnionitis/subchorionitis								X		X	X			X						
Fetal: funiculitis								X		X				X						
Other placental processes																				
Massive perivillous fibrinoid deposition																			X	
Hypoxia-ischemia-villous lesions	X	X	X	X	X	X	X	X	X	X	X	X	X	X	X	X	X	X	X	X
Chorangiosis		X		X		X	X		X		X		X					X		

Hypoxia-ischemia-villous lesions, including collapsed intervillous spaces, small villi with fibrous axes, chorangiosis, and syncytial nodes, were found in all of our placentas.

Three newborns of positive mothers tested positive for the SARS-CoV-2 RT-PCRs (15%) in their urine and stool. The corresponding placentas were also positive, including the one from the mother with HELLP syndrome.

## Discussion

To our knowledge, this is the first African study addressing placental pathology associated with infection by SARS-CoV-2 (COVID-19). The most significant finding of our study is the increased rate of lesions due to maternal vascular malperfusion (80%), formerly referred to as maternal vascular perfusion. These lesions are generally associated with oligohydramnios, intrauterine growth restriction (IUGR), prematurity, and intrauterine fetal death [2]. Maternal hypercoagulability and maternal hypertensive disorders, notably gestational hypertension and preeclampsia, constitute the main risk factors for maternal vascular malperfusion [5,8-10]. Only two patients in our study were hypertensive. Shanes et al. described a high rate of maternal vascular malperfusion lesions in 16 placentas from mothers infected with SARS-CoV-2 [2]. One of them was hypertensive. However, Komine-Aizawa et al. observed maternal vascular malperfusion lesions in only five cases (25%) among the 20 placentas studied [4]. These included one case of severe arterial hypertension and two cases presenting with severe preeclampsia [10].

Our placentas exhibited a high rate of intervillous thrombosis (IVT) (85%). This result remains higher than those reported in the literature, notably in the studies by Shanes et al. [2], Schwartz et al. [7], Smithgall et al. [11], and Baergen and Heller [5], which reported IVT rates of 37.5%, 37%, 33%, and 15%, respectively. This lesion is defined as an area of thrombosis located within the intervillous space, displacing adjacent trophoblastic villi and exhibiting parallel lamellae (lines of Zahn) [7]. It may be located in contact with the chorionic plate (subchorionic), within the central parenchyma, or in contact with the basal plate (basal). When subchorionic, it is often much more extensive, extending along the chorionic plate. It is referred to as a massive hematoma when it measures ≥1 cm in thickness and occupies ≥50% of the surface of the chorionic plate. The latter is composed of maternal blood and is associated with a high rate of fetal morbidity and mortality. Its pathogenesis remains poorly understood [6].

Thromboembolic disorders reported in association with COVID-19 may explain a state of hypercoagulability in response to the virus [2,12-14].

Lesions of accelerated villous maturation (AVM) were recorded in 12 cases (60%). These lesions are characterized by the alternation of areas of agglutinated villi with syncytial nodules and intervillous fibrin and areas of villous rarefaction. When these areas of villous rarefaction involve more than 30% of the distal villi, this is referred to as distal villous hypoplasia [8]. This phenomenon primarily reflects AVM. Our results remain higher than those reported in the literature, notably in the studies by Smithgall et al. [11] and Shanes et al. [2], which reported rates of 16% and 12.5%, respectively.

We also report villous infarctions in seven cases (35%). This percentage is close to that observed in the study by Shanes et al. (26%) [2]. The study by Smithgall et al. reports a percentage of 12% [11]. Infarctions constitute a group of placental lesions with a characteristic of maternal vascular malperfusion (MVM) [14]. They are defined as areas of ischemic coagulative necrosis of the placental parenchyma. This necrosis is due to the vasculopathy of the utero-placental artery, leading to a reduction in blood flow [15]. In the series by Ng et al., only one case among seven women infected with SARS-CoV-2 in Hong Kong presented an extensive villous infarction [13]. Microthrombi and the occlusion of placental spiral arteries, secondary to SARS-CoV-2-induced hypercoagulability, may be responsible for placental infarction [13,16]. It has been suggested that chronic changes related to MVM, such as infarction and distal villous hypoplasia, essentially require time to develop and therefore may not be observed in placentas infected with SARS-CoV-2 during the acute phase [16,17].

Decidual arteriopathy is, by definition, a pathology of the maternal vessels, which may affect the utero-placental arteries or the arterioles of the free placental membrane [7,8]. It belongs to the group of maternal vascular malperfusion (MVM) lesions and was recorded in only two cases of preeclampsia (10%). This result is consistent with the studies by Schwartz et al. (7.35%) [7] and Smithgall et al. (5.9%) [11]. A higher rate (46%) was reported by Shanes et al. [2]. Decidual arteriopathy is characterized by lesions of atherosis and the fibrinoid necrosis of the utero-placental arteries, associated with microcalcifications. Atherosis and the mural hypertrophy of the arterioles of the free placental membrane are also manifestations of maternal hypertension [14]. Yan et al. suggest that atherosis is observed in approximately 10%-40% of pregnancies complicated by early-onset preeclampsia and is also observed, but less frequently, in other maternal malperfusion syndromes such as essential hypertension and intrauterine growth restriction (IUGR) [15].

Basal decidual hematoma (retroplacental hematoma), which is also an MVM-type lesion, was observed only in patients with severe preeclampsia associated with lesions of decidual arteriopathy and intrauterine fetal death (IUFD) in 5% of cases. This result is consistent with the study by Shanes et al. [2] and the cohort of Schwartz et al. [7]. It is defined as a retroplacental hemorrhage occurring in the subplacental decidua. It is secondary to the rupture or thrombosis of a utero-placental artery [7]. Only Shanes et al. reported a case of IUFD associated with a basal decidual hematoma [2]. The association between basal decidual hematoma and thrombophilia has been described in earlier studies [8]. However, no cases of thrombophilia were observed in our study.

In contrast to MVM lesions, lesions of the fetal vascular network were described in only six cases (30%). The study by Schwartz et al. reports a percentage of 10.29% [7]. They are defined as thromboses of the vessels of the umbilical cord, chorionic plate, and stem villi, associated with vascular ectasia, endothelial edema, and the extravasation of red blood cells. Late intraluminal calcifications are also observed [10]. In contrast to our study, Komine-Aizawa et al. found that fetal vascular malperfusion lesions were the most frequent and were recorded in 45% of cases [4]. These lesions corresponded to intramural fibrin deposits, foci of vascular stromal karyorrhexis in villi, and very recent nonocclusive intramural thromboses.

As SARS-CoV-2 is a virus, it can be expected to induce inflammation [10]. Chronic inflammatory pathologies (CIP), and in particular chronic villitis, may be directly caused by certain viral infections, such as cytomegalovirus, varicella-zoster virus, and herpes simplex virus [2]. However, in our study, as in those by Shanes et al. [2] and Baergen and Heller [5], where the rates of inflammatory lesions were 18.75% and 25%, respectively, the inflammatory pathology was acute and less marked in cases of chorioamnionitis and funiculitis. Although systemic inflammation is still influenced by the SARS-CoV-2 virus, neither acute inflammatory pathology (AIP) nor CIP increased significantly. In contrast to a recent study by Ng et al., among 11 placentas from women infected with SARS-CoV-2, inflammatory and thrombohemorrhagic lesions were frequent, including four cases of chronic villitis and one case of chronic histiocytic intervillositis [13]. None of these five patients had comorbidities that could explain the placental inflammation. Patberg et al. also described a higher incidence of chronic villitis (19%) compared to a control group (4%) [16]. Smithgall et al. observed this inflammatory pathology in only one case (4%), in the form of chronic villitis [11]. No cases of chorioamnionitis were reported either by Singh et al. [1] or by Ng et al. [13].

In our series, as well as in the study by Baergen and Heller, only one case of massive perivillous fibrin deposition was reported [5]. This lesion is thought to be due to maternal hypoxia related to COVID-19 [12]. Ng et al. reported an increase in perivillous and subchorionic fibrin [13]. However, the study by Shanes et al. reported a rate of 18.75% [2]. In contrast to the studies by Zaigham et al. [9] and Schwartz et al. [7], which found much higher rates (93%), other studies suggest that coronavirus may disrupt the coagulation cascade and lead to fibrin clot formation [1,2].

Our study also included three placentas from fetuses with intrauterine fetal death (IUFD) in mothers infected with SARS-CoV-2. Histological examination revealed multiple infarctions. In one case, a basal decidual hematoma and decidual arteriopathy were associated with these infarctions, whereas in the other two, multiple intervillous thromboses were observed. Shanes et al. reported only one case of IUFD presenting with villous edema and a basal decidual hematoma on the morphological examination of the placenta. This suggests that COVID-19 may have deleterious effects on the fetus in utero [2]. Although previous studies have attributed disturbances of the coagulation cascade to coronavirus, it is currently impossible to determine whether these maternal systemic vascular alterations are directly responsible for the histological lesions of maternal vascular malperfusion (MVM) or whether they are due to an underlying pathology. The direct causal relationship therefore warrants further investigation [15,16].

Lesions of villous ischemia-hypoxia were described in all cases (100%). Considered impact lesions, they are characterized by the presence of collapsed intervillous spaces, small poorly vascularized villi, an excess of syncytiotrophoblasts, and chorangiomatosis [2]. Among these lesions, chorangiomatosis is also noted, an abnormality of the villous capillary network. It is defined by the presence of at least 10 villi containing at least 10 capillaries in at least 10 fields of terminal villi, across different regions of the placenta. It is associated with decreased maternal oxygen saturation and is more frequently observed in diabetic women. Singh et al. reported an increase in syncytiotrophoblasts and villous agglutination, which may result from utero-placental malperfusion [1]. The association between coronavirus-related respiratory involvement and maternal hypoxia suggests an increase in thromboembolic disorders associated with this virus [17,18].

In our study, chorangiomatosis was observed in 40% of cases. This rate is higher than those reported in the literature, notably in the studies by Shanes et al. [2] and Smithgall et al. [11], which reported prevalences of 25% and 12%, respectively. This difference may be explained by the lower oxygen saturation levels associated with SARS-CoV-2 infection in our positive patients [19].

SARS-CoV-2 infects tissues via its receptor, angiotensin-converting enzyme 2 (ACE2), and its cellular entry requires the cleavage of the spike protein by the serine protease TMPRSS2 [19,20]. Infection may occur via receptors expressed on the surface of the villous syncytiotrophoblast or through the disruption of the trophoblastic membrane of the villous stroma, thereby allowing the virus to access stromal cells, including resident macrophages known as Hofbauer cells [18]. The infection of the extravillous trophoblast (EVT) has also been demonstrated. Vertical transmission may occur when the virus reaches the fetal circulation through direct placental infection, fetal ingestion, or the aspiration of infected amniotic fluid [20].

In our study, only three placentas (15%) from mothers infected with SARS-CoV-2 showed a positive RT-PCR result for SARS-CoV-2 viral proteins. Conversely, in the series reported by Zaigham et al., placental tissue was positive in 100% of cases [9]. However, in the series by Schwartz et al. [7], as well as in other studies, RT-PCR for SARS-CoV-2 on placental tissue was negative in all cases [4,18,20].

In our study, RT-PCR testing for SARS-CoV-2 was positive in urine and stool samples from 10% of newborns (i.e., two newborns). These results are consistent with those reported by Zaigham et al., who observed a neonatal positivity rate of 14.28% [9]. In contrast, the studies by Shanes et al. [2] and Schwartz et al. [7] reported no SARS-CoV-2-positive newborns.

Vertical transmission is very rare. It appears that placental morphological changes, if induced by SARS-CoV-2, are related to maternal infection and inflammation rather than fetal infection. However, even in a newborn testing negative for SARS-CoV-2, abnormal pathological findings have been reported on placental examination [4]. The main receptor for SARS-CoV-2 is thought to be angiotensin-converting enzyme 2 (ACE2) [20]. This enzyme is highly expressed in cells at the maternofetal interface, such as the syncytiotrophoblast, cytotrophoblast, endothelial cells, and vascular smooth muscle cells of primary and secondary villi. However, another route of transplacental transmission cannot be excluded. Pathological examination has shown that the syncytiotrophoblast is often infected by coronavirus, in contrast to the fetus, which is not systematically infected. These findings suggest the existence of a placental barrier, although it is not fully effective [18,19]. As the molecular mechanisms of the intrauterine vertical transmission of SARS-CoV-2 have not yet been determined, the intensive clinical examination of newborns, as well as the careful morphological study of the placenta, is strongly recommended [20].

This study has several limitations: the absence of a control group; the heterogeneous grouping of symptomatic, asymptomatic, and convalescent patients; the small sample size; and its retrospective design, which limits the analysis to a descriptive level without establishing causality. The lack of long-term maternal and fetal follow-up, as well as incomplete neonatal data, limits clinicopathological correlations. Moreover, the absence of fetal autopsy prevents the exclusion of other etiologies, and RT-PCR testing alone does not definitively confirm neonatal infection or vertical transmission.

## Conclusions

A variety of viral infections during pregnancy are associated with pathognomonic placental histopathological lesions, unlike COVID-19, where no specific characteristic is identified. We have reported placental morphological lesions in 20 pregnant women infected with SARS-CoV-2 or with a history of this viral infection. High rates of MVM lesions, such as intervillous thrombosis, accelerated villous maturation, and infarcts, suggesting abnormal maternal circulation, have been noted. These results are generally concordant with those of the literature and justify close pregnancy monitoring. Furthermore, the long-term SARS-CoV-2 effects on maternal and infant health require further study.
